# Digital Management of Early-Onset Type 2 Diabetes: Empowerment, Challenges, and Future Outlook

**DOI:** 10.1007/s11892-026-01633-6

**Published:** 2026-07-10

**Authors:** Li-Jun Guo, Kah-Seng Low, Sok-Kun Tae, Lee-Ling Lim

**Affiliations:** 1 Department of Medicine, Faculty of Medicine, Universiti Malaya, Kuala Lumpur, 50603 Malaysia; 2 Department of Pediatrics, Faculty of Medicine, Universiti Malaya, Kuala Lumpur, 50603 Malaysia; 3 Department of Medicine and Therapeutics, The Chinese University of Hong Kong, Hong Kong SAR, China; 4 Asia Diabetes Foundation, Hong Kong SAR, China; 5 Baker Heart and Diabetes Institute, Melbourne, VIC Australia

**Keywords:** Early-Onset Type 2 Diabetes, Digital Health, Digital Therapeutics, Continuous Glucose Monitoring, Artificial Intelligence, Empowerment

## Abstract

**Purpose of Review:**

Early-onset type 2 diabetes (EOT2D), defined as a diabetes diagnosis before 40 years of age, is rising globally and associated with an aggressive disease course and early complications. This review examines the role of digital health technologies (DHT) in addressing the unique clinical and life-course challenges of EOT2D.

**Recent Findings:**

DHT, including continuous glucose monitoring, mobile health applications, digital therapeutics, telemedicine, remote patient monitoring, wearable devices, and artificial intelligence–based analytics, have demonstrated modest improvements in glycemic control, weight management, and patient engagement in people with type 2 diabetes. However, evidence in adults with EOT2D remains limited. Compared with usual-onset T2D, people with EOT2D may derive particular benefits due to higher digital literacy, greater lifestyle variability, and longer anticipated disease duration.

**Summary:**

Although DHT shows promise for improving empowerment and care integration in EOT2D, important gaps persist, including a lack of EOT2D-specific trials, digital divide–related inequities, interoperability challenges, and reimbursement barriers. Future research should prioritize tailored interventions and hybrid care models to optimize long-term outcomes in this high-risk population.

**Supplementary Information:**

The online version contains supplementary material available at 10.1007/s11892-026-01633-6.

## Introduction

Type 2 diabetes (T2D), once considered a disease of middle and older age, is increasingly diagnosed in adolescents and young adults. Data from the Global Burden of Disease study and the International Diabetes Federation indicate that the number of people living with diabetes now exceeds 590 million, with 75% living in low- and middle-income countries [1, 2]. Notably, the incidence of T2D in adolescents and young adults has risen disproportionately in low- and middle-income countries, which now account for nearly 80% of early-onset type 2 diabetes (EOT2D) cases globally. Among adolescents and young adults aged 10–24 years, the global incidence of type 2 diabetes increased from 56 per 100,000 in 1990 to 123.9 per 100,000 in 2021 [3]. 

EOT2D is generally defined as T2D diagnosed before the age of 40 [4]. It represents a growing public health challenge and is characterized by an aggressive disease phenotype, including accelerated β-cell dysfunction, severe insulin resistance, and a high prevalence of coexisting cardiometabolic risk factors [4]. Compared with usual-onset T2D, people with EOT2D experience more rapid progression of microvascular and macrovascular complications, leading to increased lifetime cardiovascular-kidney risk, healthcare resource utilization, and premature mortality [2, 5, 6]. 

Current management of EOT2D is largely extrapolated from studies in older adults, with minimal evidence to guide optimal treatment paradigms specific to young populations. Clinical studies indicate that metformin monotherapy as first-line treatment often fails to sustain glycemic control in EOT2D, necessitating earlier combination therapy and intensive interventions [7]. Of note, reproductive health needs, transitional care gaps from pediatric to adult services, and health-system misalignment further undermine effective management of people with EOT2D.

**Fig. 1:**
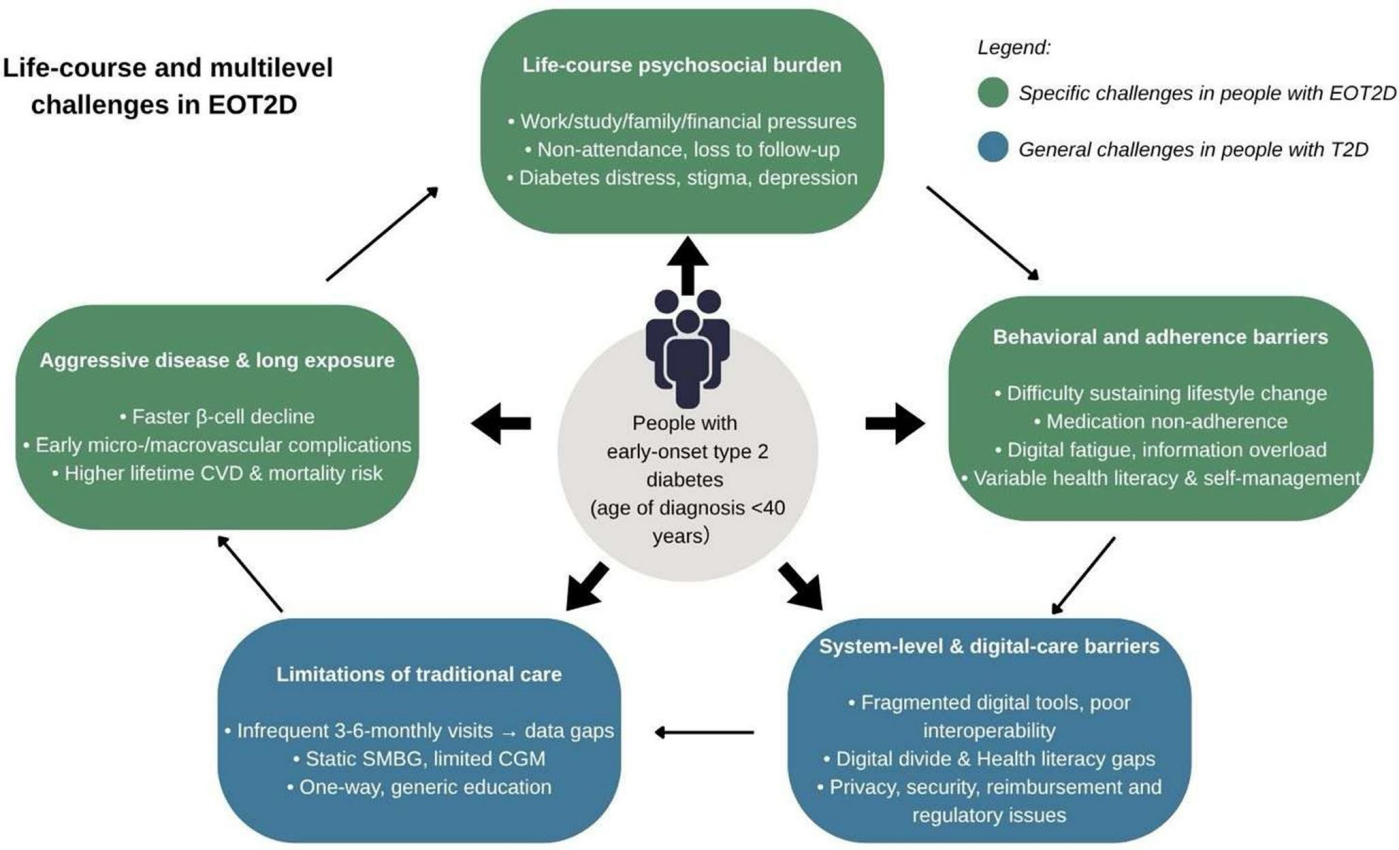
The Multifaceted Management Challenges of Early-Onset Type 2 Diabetes. CGM, continuous glucose monitoring; CVD, cardiovascular disease; SMBG, self-monitoring blood glucose

While all young adults face challenges, people with EOT2D are typically burdened by a sudden, disruptive diagnosis and the profound weight-related stigma of the disease as they are navigating critical life stages involving career establishment, family formation, and high financial pressures. These psychosocial, professional, and familial responsibilities significantly affect the adherence to and sustainability of long-term T2D management. These intersections of clinical and contextual barriers highlight the need for strategies tailored to the metabolic and life-course challenges of people with EOT2D (Fig. 1) [8].

Against this backdrop, digital health technology (DHT) has the potential to address the aforementioned care gaps in people with EOT2D [9–12]. By integrating sensing technology, mobile computing, data analytics, and remote communication. DHT enables continuous disease state monitoring, real-time behavioral interventions, and efficient patient-provider communication [9–12]. This review systematically examines core DHT applied to people with EOT2D alongside its clinical evidence, pathways for engagement and empowerment, and future directions.

### DHT: Types and Functions

DHT encompasses mobile health (mHealth) applications, telemedicine and remote monitoring platforms, continuous glucose monitoring (CGM), and regulatory-approved digital therapeutics (DTx). These tools typically integrate functions including medication reminders, dietary and physical activity tracking, educational content delivery, online messaging/video follow-ups, and automated decision support [13]. 

**Fig. 2:**
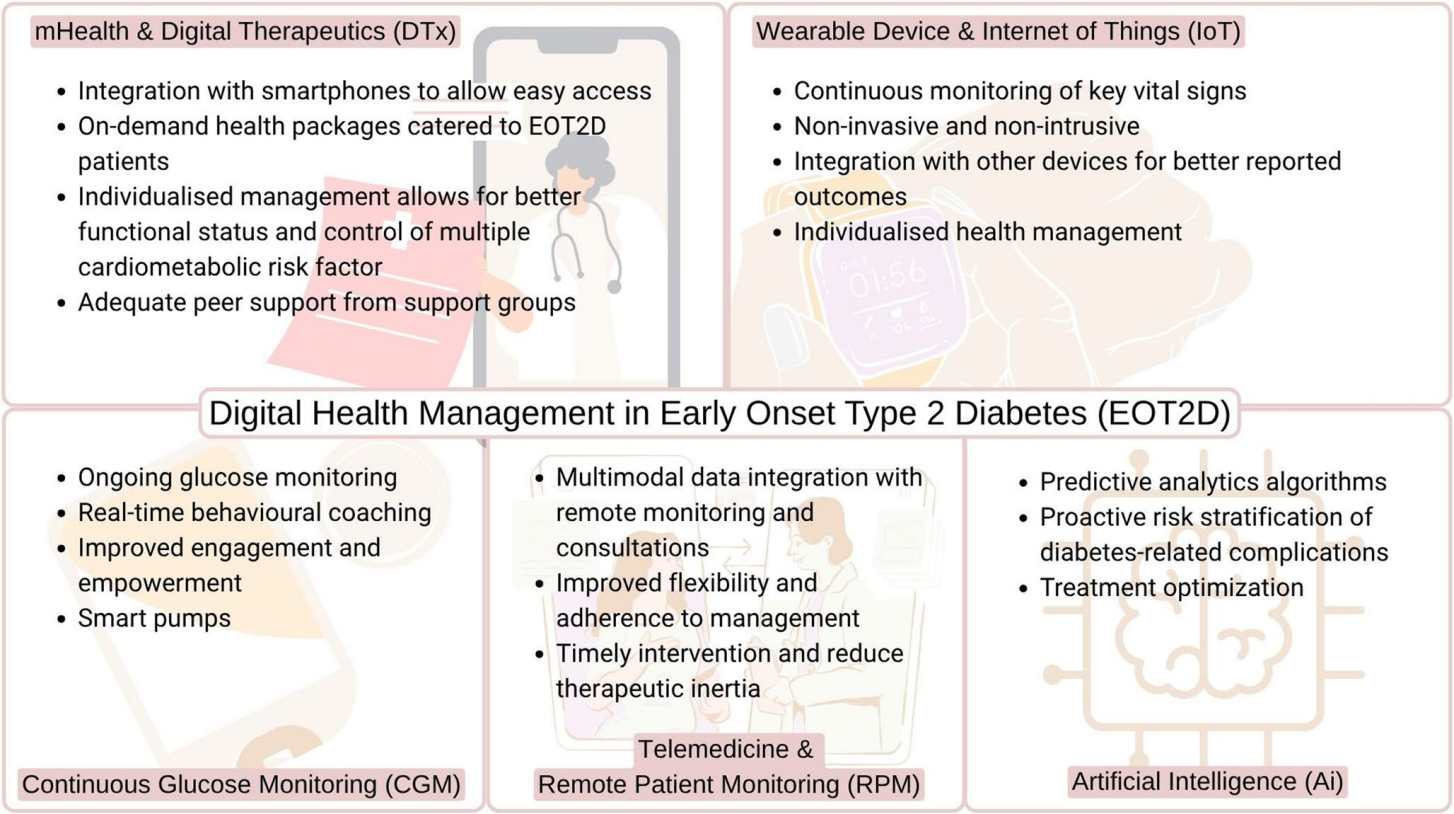
A Framework for Digital Health Management of Early-Onset Type 2 Diabetes. This framework of Digital Health Management in EOT2D comprises 5 distinct Digital Health Technologies (DHT). These DHTs work synergistically to provide high-quality, interoperable, and tiered digital care models for EOT2D patients

DHT is transforming the management of EOT2D by improving empowerment and optimization of care, as shown in Table 1. Personalized education delivered through mobile/web apps, messaging, and online platforms enhances health literacy, while visualization of integrated data on cardiometabolic risk factors, diet, physical activity, and sleep patterns supports self-monitoring and adaptive behavioral change. DHT also strengthens empowerment via remote consultations, asynchronous communication, and peer support, facilitating shared decision-making and sustained engagement (Fig. 2) [14]. Compared with conventional care models, integration of DHT with electronic health records allows prioritization of high-risk people and more efficient monitoring, with evidence suggesting improved glycemic outcomes and favorable cost-effectiveness [15]. Notably, emerging data from EOT2D populations indicate that internet-based care, SMS-based support programs, and CGM-supported management can improve glycemic control, treatment adherence, and patient engagement, further underscoring the potential of DHT-enabled, integrated care models for people with EOT2D [16–18]. Taken together, these strategies highlight how DHT can address both the clinical intensity and life-course challenges of people with EOT2D. However, DHT is not a monolithic intervention. Its impact depends on the modality, level of integration, and intended function within the care pathways. The following section examines five distinct categories of DHT, outlining their strategies, evidence to date, and relevance to the management of EOT2D.

**Table 1:** Clinical Studies and Evidence of Digital Health Technologies in the Management of Early-Onset Type 2 Diabetes

No.	Study / Year	Digital Health Technology	Country	Study Design and Population	Sample Size	Core Functional Modules	Main Outcomes	Key Findings and Implications
1	Li et al., 2025, Internet-based management study [16]	Internet-based comprehensive management system plus CGM	China	12-months RCT; adults 18–40 years, newly diagnosed T2D	120	CGM upload, personalized lifestyle advice, online education, remote care team	Lower HbA1c, better β-cell function, higher remission vs. usual care	Early intensive, internet-based follow-up can markedly improve glycemia and remission in young adults with T2D.
2	Middleton et al., 2021 (TEXT2U) [17]	Enhanced SMS reminders/support	Australia	12-months RCT; young-onset T2D(onset 18–40 years)	40	Visit reminders, lifestyle tips, self-management prompts	Greatly improved clinic attendance; no significant HbA1c change	Low-intensity SMS boosts engagement but is insufficient alone for glycemic improvement.
3	Patel et al., 2025 [18]	Real-time CGM	USA	12-weeks pilot RCT; youth-onset T2D (10–25 years)	28	CGM wear, trend display, alarms, data review	Feasible/acceptable use; no short-term HbA1c benefit; skin issues common	CGM is workable for youth T2DM but requires strategies to improve wearability, reduce stigma, and extend use.
4	Shah et al., 2025 [25]	Intermittently scanned CGM	USA	12-month cohort; publicly insured youth < 20 years with T2D	30	Provision/prescription of CGM within routine visits	CGM use declined; HbA1c remained high, TIR fell over time	Sustained CGM use in disadvantaged youth is challenging; additional behavioral and structural support is needed.
5	Khavere et al., 2025 [31]	Mixed digital and non-digital self-management interventions	Multiple	Systematic review/meta-analysis; adults 18–45 years with T2D	10 trials	Education, monitoring, behavior-change techniques via clinics, web, apps, SMS	Overall changes in HbA1c and other outcomes were small and non-significant	Current self-management interventions for younger adults show limited effect, highlighting need for stronger, age-specific digital programs.
6	Kerr et al., 2024 [37]	Apps, connected meters, remote coaching	Multiple	Systematic review/meta-analysis; adult T2D	28 trials	Data upload, education, remote coaching and feedback	Mean HbA1c reduction ~ 0.3%; greatest with high-intensity coaching	High-intensity, personalized digital coaching models are most promising and can inform EOT2D intervention design.
7	Sim et al., 2024 [38]	Digital information channels (web, social media)	Singapore	Qualitative study; young-onset T2D, 22–39 years	21	Exploration of preferred DSME formats and content	Preference for credible, accessible, individualized, empathetic education	User expectations point to key design principles for youth-oriented digital DSME platforms.
8	Misra et al., 2025 Series review [60]	Narrative review of mHealth, CGM, telehealth	Multiple	Narrative review; T2D diagnosed < 40 years	—	Overview of technology-based and other treatments	Very few trials target EOT2D directly; technology appears promising but evidence is sparse	High-quality EOT2D-specific trials of mHealth, CGM and remote multidisciplinary care are urgently required.

We searched the PubMed database covering the past five years (2021–2025) and screened relevant literature using keywords such as ‘type 2 diabetes’, ‘digital therapies’, ‘mobile applications’, ‘mobile health’, ‘telemedicine’, and ‘self-management’. Priority was given to studies that provided a detailed description of structured digital interventions and reported clinical or patient-relevant outcomes

### Continuous Glucose Monitoring (CGM)

CGM technology employs subcutaneous sensors to monitor interstitial fluid glucose concentrations, providing real-time dynamic glucose profiles [19, 20]. Compared with conventional fingerstick glucose monitoring, a 2024 meta-analysis of 12 randomized clinical trials reported a 0.3%–0.4% reduction in HbA1c and a 6% improvement in time-in-range (TIR) with use of CGM in people with T2D treated with insulin therapy and/or oral glucose-lowering drugs [19]. People with T2D who experience greater lifestyle variability and a higher risk of nocturnal hypoglycemia and hypoglycemia unawareness will likely benefit from CGM [21]. 

CGM allows personalized, real-time feedback that helps young adults visualize how diet, physical activity, sleep, stress, and medication adherence influence their daily glycemic support shared decision-making, and enable remote titration of therapies when CGM data are integrated with telemedicine platforms and electronic health records, thereby positioning CGM as a central “data spine” that underpins other DHT for people with EOT2D [22]. Emerging multimodal deep learning models that fuse real‑time CGM and clinical data can now forecast interstitial glucose 15–60 min ahead with clinically acceptable accuracy, creating a window for algorithms to anticipate glycemic excursions rather than merely record them [23]. For people with EOT2D, such predictive engines could be embedded into “AI dietitian”–style tools that deliver culturally-tailored prompts about diets, physical activity, and medication, shifting digital health from passive monitoring to proactive risk management across home, school, and workplace settings. To translate these “AI dietitian” systems into routine care, models will need training on diverse, high‑quality datasets, rigorous clinical validation, and localization to local foods, languages, and care pathways in close collaboration with clinicians and dietitians [24]. 

Notably, existing studies in EOT2D are limited by small sample sizes, with pilot randomized and feasibility studies reporting short-term improvements in glycemic profiles and acceptability of CGM among publicly insured or clinic-based EOT2D [18, 25]. This paucity of large-scale data is driven even more profoundly by the unique socio-demographic and behavioral barriers inherent to EOT2D. People with EOT2D often present with higher rates of socio-economic deprivation, mental health conditions, and competing life demands compared with those with usual-onset T2D (Fig. 1) [26, 27]. These factors culminate in high default rates. Taken together, the small sample sizes in DHT studies reflect not merely a limited epidemiological pool, but a highly vulnerable, “hard-to-reach” population that traditional diabetes care persistently struggles to engage.

While initial uptake of CGM may be encouraging, sustained use often declines over time because of device burden, skin issues, costs, and access barriers [25]. These observations underscore that, although CGM can provide the data backbone for many DHT applications, specific strategies are needed to support long‑term acceptability and continuous use in people with EOT2D [25]. In a population facing long disease duration and elevated lifetime risk of cardio-renal events, such continuous, context-rich monitoring offers a plausible pathway to better cardiometabolic risk factor control and clinical outcomes, although head-to-head comparisons of CGM efficacy and hard endpoints (hospitalization, cardiovascular and kidney events, mortality) between people with EOT2D and usual-onset T2D remain limited [28]. 

### Wearable Devices and the Internet of Things (IoT)

Wearable devices such as smartwatches and fitness trackers can continuously monitor heart rate, sleep patterns, step counts, and activity intensity, providing objective data for lifestyle assessment and behavioral nudges. IoT architectures enable these devices to connect with CGM, smart insulin pens, and home blood pressure or weight monitors, forming an integrated personal health ecosystem. In adults with T2D, wearable technology-based physical activity interventions significantly increase daily step counts and often improve systolic blood pressure, body weight, and other cardiometabolic risk factors, with small but favorable effects on glycemic control [29]. To date, there is a lack of interventional studies specifically evaluating wearable- or IoT-based programs in people with EOT2D, and no robust data on hard clinical endpoints such as hospitalization and cardiovascular-kidney events in this population [29]. While wearables and IoT-enabled devices such as CGM and sensor-equipped monitoring platforms have been studied in T2D populations, there is a notable paucity of research focusing exclusively on people with EOT2D. 

Despite this evidence gap, the potential benefits of wearables and IoT for EOT2D are high. People with EOT2D frequently face socioeconomic, work, study, and family pressures that make it challenging to prioritize regular physical activity [29]. However, because this younger population is highly digitally connected, they are uniquely receptive to smartphone‑based support [29]. By capturing physical activity and sleep patterns in real-time, synchronizing these data with glucose and blood pressure readings, and delivering timely feedback, reminders, and goal-setting support, wearable-centric interventions could help people with EOT2D understand their personalized glycemic responses to physical activity, lower the cognitive burden of self-monitoring, and enable more realistic, adaptive physical activity prescriptions. IoT devices may serve as practical tools for empowerment in people with EOT2D, by enhancing self-efficacy, healthy behaviors, and shared decision-making needed to confirm these hypothesized benefits.

### mHealth Applications and Digital Therapeutics (DTx)

Smartphone applications currently represent the most prevalent form of DHT in diabetes care. mHealth apps typically combine self-monitoring of blood glucose, dietary, and physical activity with data visualization, personalized reminders, educational modules, goal setting, and in-app communication with healthcare providers, while digital therapeutics (DTx) deliver clinically validated, software-based interventions for disease prevention and management [30]. A 2024 systematic review of 28 digital interventions for adults with T2D (including 23 randomized controlled trials) indicate that structured diabetes management applications, particularly those incorporating personalized feedback, goal setting, and patient-provider communication features, can improve HbA1c, dietary behaviors, and physical activity levels in people with T2D [31]. 

Direct evidence for mHealth and DTx specifically in people with EOT2D remains limited, as the majority of digital nutrition and lifestyle programs were evaluated in general adults with T2D rather than in those with EOT2D or in head‑to‑head comparisons with usual‑onset T2D. A typical example is the Virta Health remote continuous care model, developed by Virta Health Corp., which integrates app-based low-carbohydrate nutritional therapy, continuous remote biomarker monitoring, and health coaching delivered by a multidisciplinary clinical team [32]. A non-randomized controlled study of adults with T2D was conducted and had successfully achieved a mean HbA1c reduction of 1.3% (from 7.6% to 6.3%) and a mean weight loss of 12% at one year, with sustained improvements observed at two years [33]. However, the study population was not restricted to those with EOT2D, and the lack of age-specific reporting precludes a better understanding of the younger group. Similarly, digital lifestyle programs such as Omada Health, which combine structured online educational curricula, remote health coaching, and peer support, have demonstrated sustained weight loss in large-scale digital diabetes prevention programs [34]. For example, a web-based intervention reported a mean weight loss of 4.7% at 12 months, with durability over longer (up to 2 years) follow-up. However, these studies primarily involved people with prediabetes rather than established EOT2D, and glycemic outcomes were not the primary endpoints [34]. These findings suggest that intensive, coach‑supported digital interventions have strong potential to empower people with EOT2D, by providing flexible, high‑touch support that fits around work and family commitments. Future studies examining their implementation strategies to sustain long-term effectiveness in people with EOT2D are required [35]. 

### Telemedicine and Remote Patient Monitoring (RPM) Platforms

Telemedicine facilitates remote consultations via video, telephone, or asynchronous messaging, offering a way to deliver specialist input without requiring frequent in‑person visits. People with EOT2D often have to juggle multiple commitments, including work and family, on top of requiring frequent clinic visits. Hence, the flexibility of telemedicine and RPM platforms is particularly attractive, as it significantly reduces the physical and psychological challenges to engaging in long-term diabetes care. RPM platforms further enable them to automatically upload data from CGM, smart blood glucose meters, and wearable devices to secure cloud systems, allowing healthcare professionals to remotely review trends and intervene appropriately [36].

Compared with in-person clinical visits, telemedicine care can improve glycaemia in young and middle-aged people with T2D and overweight/obesity, with several remote coaching and RPM programs demonstrating modest HbA1c reductions and improved treatment adherence in younger adults [31, 37–39]. When RPM is integrated with clinical decision support systems, clinicians can rapidly identify people with suboptimal risk factor control or at increased risk of hypoglycemia and those with hypoglycemia unawareness. This enables earlier, more precise treatment adjustments and reduces healthcare utilization, including emergency department visits [40, 41]. In a large digital diabetes RPM program, 63% of participants with poor baseline control reached target glucose or HbA1c on a stable oral and/or injectable regimen, and a 2022 meta-analysis of 20 randomized controlled trials reported that RPM lowered HbA1c by 0.4% over 6 months, supporting earlier, more precise treatment adjustments and better risk factor control [42, 43]. Future studies need to move beyond short‑term feasibility towards hybrid diabetes care models that systematically combine telemedicine, RPM, and in‑person clinic visits. Further evaluations of such models to sustain engagement, improve metabolic and psychosocial outcomes, and narrow access gaps for people with EOT2D are also required [44–46]. 

### Artificial Intelligence and Predictive Modelling

AI algorithms can be applied to the large, high‑frequency datasets generated by CGM, wearables, and mHealth apps to predict short‑term glycemic trajectories, identify high‑risk patterns such as nocturnal hypoglycemia, and provide personalized dietary, physical activity, and treatment recommendations. Multiple reviews and methodological studies show that machine‑learning and deep‑learning models improve predictive accuracy for hypoglycemia, hyperglycemia, and overall glycemic variability compared with traditional approaches, laying the groundwork for data‑driven decision support and ultimately, closed‑loop systems in T2D [47–49]. Adult-onset T2D generally presents with less glycemic variability than T1D. People with EOT2D have an aggressive disease trajectory, rapid pancreatic beta-cell dysfunction, and postprandial glycemic excursions [4]. These are mainly driven by complex behavioral and socio-environmental factors rather than absolute insulin deficiency. The incremental benefit of AI in people with EOT2D extends beyond the optimization of automated insulin delivery (AID) systems. While AID in people with T1D mainly focuses on reactive or short-term predictive insulin dosing, AI applications in those with EOT2D integrate multimodal data, including CGM metrics, dietary intake, physical activity, and medication adherence. Hence, AI in EOT2D shifts the paradigm from algorithmic insulin titration to holistic, preventative risk management that addresses the multifactorial drivers of EOT2D. In addition, among people with EOT2D who face a longer lifetime exposure to hyperglycemia and are at risk of cardiovascular-kidney complications and premature death than those with usual‑onset T2D, such anticipatory tools are particularly relevant because they enable earlier, more precise risk detection and support proactive treatment optimization over decades of disease course [4, 50, 51]. 

Early clinical and real‑world studies show that AI‑driven personalized nutrition and image recognition-based food‑logging apps can reduce postprandial glycemic excursions, improve glycemic variability, and reduce the burden of manual dietary recording. These can enhance empowerment and self-efficacy in people with T2D [52, 53]. However, most existing models have been developed in small or narrowly defined cohorts and rarely include clearly characterized EOT2D populations. Hence, rigorous external validation across diverse age groups, ethnicities, and sociocultural settings, as well as long‑term evaluation of clinical and psychosocial outcomes in people with EOT2D, should be prioritized [54]. 

Compared with usual-onset T2D, people with EOT2D tended to have a 0.5-1.0% higher HbA1c at diagnosis, greater obesity and hypertension burden, and a 2–3‑fold higher risk of microvascular and macrovascular complications at any given age [4]. A meta‑analysis of > 1.3 million people reported that, compared with usual-onset T2D, EOT2D was associated with markedly increased risks of cardiovascular disease, kidney disease, and all‑cause mortality, and that each 10‑year earlier age at diagnosis conferred 20–30% higher relative risk of major vascular events and death [55, 56]. Similarly, youth-onset cohorts such as TODAY/TODAY2 showed that by a mean diabetes duration of 13 years (early adulthood), up to 60% of participants develop at least one microvascular complication, and many have multiple complications, underscoring the need for early, intensive, and precisely targeted intervention in this vulnerable group [55, 57–59]. In this context, AI and machine learning–based predictive models, which can outperform traditional scores for predicting incident T2D and its complications, are likely to yield greater lifetime benefit in EOT2D than in usual-onset T2D by enabling earlier identification of high-risk trajectories and more aggressive preventive care over several decades of disease [55, 57, 58]. 

### Key Challenges During Implementation of DHT

The fundamental gap in the current management of EOT2D is the failure of traditional, episodic care models to mitigate the uniquely aggressive trajectory of EOT2D [28, 60]. Traditional care often suffers from high default rates and clinical inertia in this vulnerable group [28]. By leveraging real-time behavioral prompts and gamified digital platforms, DHT provides continuous engagement and empowerment to maintain lifestyle modifications between clinic visits [31, 61]. The sustained behavioral interventions facilitated by DHT could indeed delay the initiation or escalation of glucose-lowering therapies (e.g., insulin, glucagon-like peptide-1 receptor agonists) [32, 33]. This will likely yield cumulative cost savings for healthcare systems, alongside mitigating the burden of premature morbidity [56]. 

Despite promising prospects, the implementation of DHT in people with EOT2D faces multiple challenges. At the technical level, issues such as poor interoperability between different devices and platforms, a lack of unified data standards, and significant variations in system stability and user experience adversely impact long-term usage and large-scale deployment. These implementation challenges are common to DHT in general rather than being specific to EOT2D alone. Furthermore, insufficient integration with existing healthcare systems, including data security and privacy concerns, means that numerous DHT operate as isolated “information silos”. When these data cannot integrate seamlessly with electronic health record systems, this will not only increase clinicians’ workload but also hinder effective data utilization [62, 63]. Similarly, people with EOT2D live with diabetes across longer and more complex life stages. Fragmented and non‑interoperable systems may disproportionately increase their treatment burden and disrupt continuity of care when they transition from pediatric to adult services.

At the behavioral level, young people from different socioeconomic backgrounds and with varying levels of health literacy exhibit disparities in their acceptance and proficiency with DHT, with the “digital divide” potentially exacerbating existing health inequalities [64, 65]. Simultaneous operation of multiple devices and applications while managing vast amounts of data and reminders may also induce “digital fatigue” and non-adherence to treatment. Sustained long-term use of apps and devices inherently requires motivation and self-discipline. Some studies reported attenuation of intervention effectiveness over time, suggesting the need for design elements such as gamification, personalized notifications, and social support to enhance user retention [31, 61, 66]. 

At the institutional level, reimbursement policies, regulatory approvals, and liability definitions remain imperfect, constraining the clinical integration and commercial sustainability of innovative tools such as DTx. A few health economics studies reported that many countries still lack stable payment mechanisms and unified evaluation frameworks for DTx, remote monitoring, and digital follow-ups, leaving healthcare institutions facing financial and compliance uncertainties when scaling adoption [67]. Furthermore, legal and ethical norms concerning algorithmic liability, data ownership, and cross-border data flows are evolving, which have also slowed the deep integration of DHT into diabetes care pathways. Figure 2 illustrates how DHT can help address the challenges faced by people with EOT2D.

### Future Directions and Research Priorities

Future research and policy efforts should prioritize tailored, life-course–oriented interventions and hybrid care models within broader strategies for managing EOT2D at both individual and population levels [60]. Future DHT of EOT2D needs to transition from ‘tool development’ to ‘system integration’, encompassing evidence-based, person-centered, and seamlessly integrated with existing healthcare systems [68]. The future lies not in complete virtualization, but in the deep integration of online and offline care. Basic monitoring, education, and follow-up can be delivered via digital platforms, while complex diagnostics, treatment planning, and annual complication screening remain the preserve of physical healthcare settings. This model maximizes resource efficiency and the convenience of people with EOT2D [62, 69, 70]. 

From a research perspective, DHT for EOT2D currently lags behind that for usual‑onset T2D. Future randomized clinical trials and real‑world implementation studies will need to look into people with EOT2D with deliberate assessment of complication incidence, quality of life, mental health, work productivity, and reproductive health, and with subgroup analyses comparing EOT2D and usual‑onset T2D, whenever feasible [71–73]. 

During the early phases of intervention design, developers should explicitly consider the sociocultural and economic context of people with EOT2D, who are often digitally literate but may have financial instability and fluctuating engagement [55]. Priorities include multilingual content, ultra‑simplified user interfaces that minimize interaction burden, low‑cost or phone‑agnostic solutions, and partnerships with schools, workplaces, and community organizations to deliver digital literacy and peer‑support programs. These features are appealing in Asia and low‑ and middle‑income countries, where the incidence of EOT2D is rising rapidly, but DHT and health inequities remain significant [74]. 

Finally, precision‑targeted and adaptive interventions that blend behavioral science with AI-driven analytics are needed to move from pilots to sustainable scale‑up, including AI‑supported nutrition tools that are now at the proof‑of‑concept stage [75]. Digital self‑management ecosystems should build on evidence from multifeatured mobile apps and digital DSMES platforms that integrate self‑monitoring, education, coaching, and peer support and have achieved meaningful and, in some cases, HbA1c and weight reductions in adults with long‑standing T2D [76–78]. Flash and continuous glucose monitoring, interoperable data sharing systems, and nurse‑led web‑based education illustrate how integrated monitoring and remote follow‑up can enhance glycemic control, self‑care behaviors, and well‑being when embedded within routine care pathways [79–81]. Digital CBT‑based therapeutics further demonstrate that targeting psychosocial and behavioral drivers can yield additional glycemic benefits beyond standard pharmacotherapy [82]. Multi‑stakeholder collaboration platforms that extend support into home, school, workplace, and community settings, together with reimbursement mechanisms that specifically cover DHT for people with EOT2D, will be essential to adapt these models to younger, high‑risk populations. For additional context, please refer to Supplementary Table 1. Supplementary Fig. 1 illustrates how DHT can be integrated into a layered care model for people with EOT2D.

## Conclusion

The increasing burden of EOT2D calls for innovative management approaches. DHT offers a promising solution to the unique challenges faced by this population by providing personalized, supportive, and sustainable management. Their successful implementation hinges upon enhancing self-management capabilities and optimizing shared decision-making. To realize its full potential, we need to systematically address challenges across technological, clinical, economic, and societal dimensions. By establishing an evidence-based, person-centered digital ecosystem that integrates seamlessly with the healthcare systems, we can pave the way towards a healthier future for people with EOT2D.

Footnotes: We searched the PubMed database covering the past five years (2021–2025) and screened relevant literature using keywords such as ‘type 2 diabetes’, ‘digital therapies’, ‘mobile applications’, ‘mobile health’, ‘telemedicine’, and ‘self-management’. Priority was given to studies that provided a detailed description of structured digital interventions and reported clinical or patient-relevant outcomes.

## Supplementary Information

Supplementary Material 1.

Supplementary Material 2.

## Data Availability

No datasets were generated or analysed during the current study.

## Key References

- Lim LL, Jones S, Cikomola JC, Hivert MF, Misra S. Understanding the drivers and consequences of early-onset type 2 diabetes. Lancet. 2025;405(10497):2327–40.
  - This comprehensive and high-impact review delineates the epidemiology, pathophysiological mechanisms, and long-term clinical consequences of EOT2D. It establishes EOT2D as a biologically and clinically distinct phenotype characterized by accelerated β-cell failure and increased lifetime cardiometabolic risk, providing the conceptual foundation for the need for tailored and innovative management strategies, including digital health integration.
- Luk A, Wild SH, Jones S, Anjana RM, Hivert M-F, McCaffrey J, et al. Early-onset type 2 diabetes: the next major diabetes transition. The Lancet. 2025;405(10497):2313–26.
  - This landmark article frames EOT2D as a global epidemiological transition with profound implications for healthcare systems. It highlights the aggressive disease trajectory, earlier complication onset, and socioeconomic burden associated with early diagnosis, reinforcing the urgency for scalable, life-course–oriented, and technology-enabled care models.
- Jancev M, Vissers T, Visseren FLJ, van Bon AC, Serné EH, DeVries JH, et al. Continuous glucose monitoring in adults with type 2 diabetes: a systematic review and meta-analysis. Diabetologia. 2024;67(5):798–810.
  - This meta-analysis synthesizes randomized clinical trial evidence demonstrating clinically meaningful improvements in HbA1c and time-in-range with CGM use in type 2 diabetes. It provides a robust evidence base supporting real-time monitoring technologies discussed in this manuscript and their potential translational relevance for EOT2D populations.
- Liu K, Li L, Ma Y, Jiang J, Liu Z, Ye Z, et al. Machine Learning Models for Blood Glucose Level Prediction in Patients With Diabetes Mellitus: Systematic Review and Network Meta-Analysis. JMIR Med Inform. 2023;11:e47833.
  - This systematic review evaluates machine-learning and deep-learning approaches for glycemic prediction and demonstrates improved predictive accuracy over traditional statistical methods. It supports the future integration of artificial intelligence–driven decision support and precision digital interventions for individuals with EOT2D.
