# Supplementary material for: Digital Management of Early-Onset Type 2 Diabetes: Empowerment, Challenges, and Future Outlook
Source: Curr Diab Rep. 2026 Jul 10;26(1):20. doi: 10.1007/s11892-026-01633-6 (PMC13354610; doi:10.1007/s11892-026-01633-6)
Supplement: Supplementary file 1 — Supplementary Material 1. [file 11892_2026_1633_MOESM1_ESM.pdf]

SPRINGER NATURE

Change of authorship request form - Journals (pre-acceptance)

Important information. Please read.

- This form should be used by authors to request any change in authorship (adding/deleting authors) including changes in corresponding authors. This form should not be used for name changes. Please fully complete all sections. Use black ink and block capitals and provide each author's full name with the given name first followed by the family name.

- By signing this declaration, all authors guarantee that the order of the authors are in accordance with their scientific contribution, if applicable as different conventions apply per discipline, and that only authors have been added who made a meaningful contribution to the work.

- Please note, in author collaborations where there is formal agreement for representing the collaboration, it is sufficient for the representative or legal guarantor (usually the corresponding author) to complete and sign the Authorship Change Form on behalf of all authors, **next to the added/removed author(s)**. **(Complete Section 3, followed by Section 6.)**

  In author collaborations where there is no formal agreement for representing the collaboration and **there are more than 10 authors**, one may sign for all, provided the signer appends correspondence that attests that each of the authors have agreed to the change **and the added/removed authors sign the form**. **(Complete Section 3, followed by Section 6.)**

- Please note, we cannot investigate or mediate any authorship disputes. If you are unable to obtain agreement from all authors (including those who you wish to be removed) you must refer the matter to your institution(s) for investigation. Please inform us if you need to do this.

- If you are not able to return a fully completed form within **30 days** of the date that it was sent to the author requesting the change, we may have to withdraw your manuscript. We cannot publish manuscripts where authorship has not been agreed by all authors (including those who have been removed).

- Incomplete forms will be rejected.

- Please return/upload this form, fully completed, to the Journals Editorial Office. The Journal and/or Publisher will consider the information you have provided to decide whether to approve the proposed change in authorship. We may decide to contact your institution for more information or undertake a further investigation, if appropriate, before making a final decision.

SPRINGER NATURE Springer Nature is one of the world's leading global research, educational and professional publishers, created in May 2015 through the combination of Nature Publishing Group, Palgrave Macmillan, Macmillan Education and Springer Science+Business Media.

Change of authorship request form - Journals (pre-acceptance)

SPRINGER NATURE

Section 1: Please provide the current title

Manuscript ID no.: 1643d717-bf43-4d6c-985e-b103296ab5f1v2.0

Title: Digital Management of Early-Onset Type 2 Diabetes: Empowerment, Challenges, and Future Outlook

Section 2: Please provide the previous authorship, in the order shown on the manuscript before the changes were introduced. Please indicate the corresponding author by adding (CA) behind the name.

	First name(s)	Family name	ORCID or SCOPUS id, if available
1 st author	Li-Jun	Guo	
2nd author	Sok-Kun	Tae	JTD-2979-2023
3rd author	Lee-Ling	Lim	ORCiD: 0000-0002-6214-6924
4th author			
5th author			
6th author			
7th author			
8th author			
9th author			
10 th author			

Please use an additional sheet if there are more than 10 authors.

SPRINGER NATURE Springer Nature is one of the world,s leading global research, educational and professional publishers, created in May 2015 through the combination of Nature Publishing Group, Palgrave Macmillan, Macmillan Education and Springer Science+Business Media.

Section 3: Please provide a justification for change. Please use this section to explain your reasons for changing the authorship ofyour manuscript, e.g. what necessitated the change in authorship? Please refer to the (journal) policy pages for more information about authorship. Please explain why omitted authors were not originally included and/or why authors were removed on the submitted manuscript.

Kah-Seng Low provided the Figure 2. Therefore, we decided to add his name on this manuscript as appreciation of his contribution.

Section 4: Proposed new authorship. Please provide your new authorship list in the order you would like it to appear on the manuscript. Please indicate the corresponding author by adding (CA) behind the name. <u>If the CorrespondingAuthor has changed, please indicate the reason under section 3</u>.

	First name(s)	Family name (this name will appear in full on the final publication and will be searchable in various abstract and indexing databases)	Affiliated institute	E-mail address
1 st author	Li-Jun	Guo	Universiti Malaya	guolijun1117@gmail.com
2nd author	Kah-Seng	Low	Universiti Malaya	kahsenglow2001@gmail.com
3rd author	Sok-Kun	Tae (Joint CA)	Universiti Malaya	taesokkun@um.edu.my
4th author	Lee-Ling	Lim (Joint CA)	Universiti Malaya	limleeling@um.edu.my
5th author				
6th author				
7th author				
8th author				
9th author				
10 th author				

Please use an additional sheet if there are more than 10 authors.

Section 5: Author contribution, Acknowledgement and Disclosures. Please use this section to provide a new disclosure statement and, if appropriate, acknowledge any contributors who have been removed as authors and ensure you state what contribution any new authors made (if applicable per thejournal or book (series) policy).

Please ensure these are updated in your manuscript - after approval of the change(s) - as our production department will not transfer the information in this form to your manuscript.

New acknowledgements:

Not Applicable

New Disclosures (financial and non-financial interests, funding):

Not Applicable

New Author Contributions statement (if applicable per the journal policy):

Kah-Seng Low provided the Figur 2. Therefore, we decided to add his name on this manuscript as appreciation of his contributi

SPRINGER NATURE

Change of authorship request form - Journals (pre-acceptance)

Section 6: Declaration of agreement. *All*authors, unchanged, new and removed *must*sign this declaration.

(NB: Please print the form, (docu)-sign and return/upload a scanned copy. Please note that signatures that have been inserted as an image file are acceptable as long as it is handwritten. Typed names in the signature box are unacceptable.) *** Please delete as appropriate. Delete all of the bold if you were on the original authorship list and are remaining as an author.**

	First name	Family name		Signature	Date
1 st author	Li-Jun	Guo	I agree to the proposed new authorship shown in section 4 ~~/and the addition/removal*of my name to the authorship list /and the proposed change in corresponding author~~	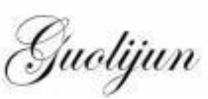	17/3/2026
2nd author	Kah-Seng	Low	I agree to the proposed new authorship shown in section 4 **/and the addition/~~removal~~*of my name to the authorship list** ~~/and the proposed change in corresponding author~~	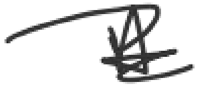	17/3/2026
3rd author	Sok-Kun	Tae	I agree to the proposed new authorship shown in section 4 ~~/and the addition/removal*of my name to the authorship list /and the proposed change in corresponding author~~	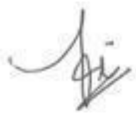	17/3/2026
4th authors	Lee-Ling	Lim	I agree to the proposed new authorship shown in section 4 ~~/and the addition/removal*of my name to the authorship list /and the proposed change in corresponding author~~	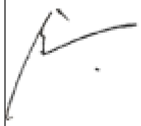	17/3/2026
5th author			I agree to the proposed new authorship shown in section 4 **/and the addition/removal*of my name to the authorship list** /and the proposed change in corresponding author		
6th author			I agree to the proposed new authorship shown in section 4 **/and the addition/removal*of my name to the**		

SPRINGER NATURE Springer Nature is one of the world,s leading global research, educational and professional publishers, created in May 2015 through the combination of Nature Publishing Group, Palgrave Macmillan, Macmillan Education and Springer Science+Business Media.

			**authorship list** /and the proposed change in corresponding author		
7th author			I agree to the proposed new authorship shown in section 4 **/and the addition/removal*of my name to the authorship list** /and the proposed change in corresponding author		
8th author			I agree to the proposed new authorship shown in section 4 **/and the addition/removal*of my name to the authorship list** /and the proposed change in corresponding author		
9th author			I agree to the proposed new authorship shown in section 4 **/and the addition/removal*of my name to the authorship list** /and the proposed change in corresponding author		
10th author			I agree to the proposed new authorship shown in section 4 **/and the addition/removal*of my name to the authorship list** /and the proposed change in corresponding author		

Please use an additional sheet if there are more than 10 authors.

In case of author collaborations with formal agreement:

	Name of consortium/consortia	First name	Family name		Signature	Date
Representative/legal guarantor				I agree to the proposed new authorship shown in section 4 **/and the addition/removal*of my name tothe authorship list** /and the proposed change in corresponding author		

Both added/removed authors should complete the information in the first table under Section 6.

--- End of form ----
