# Supplementary material for: Digital Management of Early-Onset Type 2 Diabetes: Empowerment, Challenges, and Future Outlook
Source: Curr Diab Rep. 2026 Jul 10;26(1):20. doi: 10.1007/s11892-026-01633-6 (PMC13354610; doi:10.1007/s11892-026-01633-6)
Supplement: Supplementary file 2 — Supplementary Material 2. [file 11892_2026_1633_MOESM2_ESM.docx]

**Appendix**

**Supplementary Table 1.** A Review of Clinical Research on Digital Health Technologies in Type 2 Diabetes Management

| **No.** | **Study / Year** | **Digital health technology** | **Study design and population** | **Core functional modules** | **Main outcomes (T2D-related)** | **Key findings and implications** |
| --- | --- | --- | --- | --- | --- | --- |
| 1 | Sun et al., 2023, China (AI dietitian concept) (75) | AI-based nutritionist (LLM + ingredient image recognition + WeChat mini-program) | Preclinical validation; surveys, model testing, and prototype development, no clinical outcomes yet | AI nutrition counselling and food image recognition for diet support | Demonstrated technical feasibility and strong model performance, but no HbA1c/weight data yet | AI nutrition support is promising but requires clinical trials before routine use |
| 2 | Lim et al., 2022, Singapore (DLITE cohort) (76) | “nBuddy Diabetes” mobile APP for T2D/prediabetes management | Prospective cohort (intervention arm of RCT); 171 adults with T2D or prediabetes, 6‑months follow-up​ | App-based self-monitoring, education, and remote professional support | Greater HbA1c and weight reduction with high app engagement | High engagement with multi-feature apps can meaningfully improve glycaemic and weight outcomes​ |
| 3 | Berthoumieux et al., 2024, USA (Omada for Diabetes) (77) | Digital DSMES platform (app/web + devices + coaching)​ | Real-world retrospective cohort; 1,322 adults with T2D, 12‑months follow-up​ | Online lessons, remote coaching, peer support, monitoring devices | Large A1c reduction in poorly controlled patients and maintained control in others, with modest weight loss | Comprehensive digital DSMES can achieve large, durable A1c improvements in routine care |
| 4 | Zhang et al., 2019, China (Welltang RCT) (78) | “Welltang” diabetes app with/without interactive management | Single-centre 3-arm RCT; 234 adults with T1D/T2D, 6‑months follow-up​ | App-based self-monitoring, education, patient support group and clinician communication | Best HbA1c and lipid improvements achieved with app and professional interactive management​ | Professional support on top of an app is crucial for sustained glycaemic benefit |
| 5 | Eeg‑Olofsson et al., 2023, Sweden (NDR FreeStyle Libre) (79) | FreeStyle Libre flash glucose monitoring system | National registry real-world cohort; 3,202 adults with T2D starting FreeStyle Libre, HbA1c subset n=711, 12 months follow-up | Sensor-based glucose monitoring with trend display and data sharing | Sustained HbA1c reduction, especially in insulin-treated and high-baseline HbA1c patients | Flash glucose monitoring supports better long-term glycaemic control in poorly controlled, insulin-treated T2D |
| 6 | Terkes et al., 2024, Turkey (web education RCT) (80) | Nurse-led web-based diabetes education platform | Single-centre RCT; 89 insulin-treated adults with T2D, 3‑months follow-up​ | Web education, nurse monitoring, reminders, and remote follow-up | Improved HbA1c, weight indices, and self-care versus usual follow-up | Structured web education can enhance glycaemic control and self-management in routine practice |
| 7 | Roth et al., 2025, Germany (SYSTA) (81)​ | ESYSTA interoperable digital diabetes management system | Multicentre RCT; 204 insulin-treated adults with type 1 or type 2 diabetes, 6‑months follow-up | Integrated data upload, dashboards, alerts, clinician access, tailored messaging​ | Modest additional HbA1c reduction and better well-being in adherent users​ | Integrated digital systems can add modest but relevant metabolic and psychosocial benefits when embedded in care pathways​ |
| 8 | Hsia et al., 2022, USA (BT‑001 CBT app) (82) | BT‑001 digital therapeutic app delivering CBT | Decentralized RCT; 669 adults with T2D, 90‑days primary endpoint​ | Digital CBT modules with behaviour tracking and automated feedback | HbA1c reduction of ~0.4% versus control, greater reduction in highly engaged users | Digital CBT can modestly improve glycaemic control as an adjunct to standard therapy |

Footnotes: We searched the PubMed database covering the past five years (2021-2025) and screened relevant literature using keywords such as ‘type 2 diabetes’, ‘digital therapies’, ‘mobile applications’, ‘mobile health’, ‘telemedicine’, and ‘self-management’. Priority was given to studies that provided a detailed description of structured digital interventions and reported clinical or patient-relevant outcomes. AI, artificial intelligence; CBT, cognitive behavioral therapy; DSMES, diabetes self-management education scheme; LLM, large-language model; RCT, randomized clinical trial; T2D, type 2 diabetes.

**Supplementary Figure 1:** An Integrated Digital Healthcare Ecosystem for Early-Onset Type 2 Diabetes.


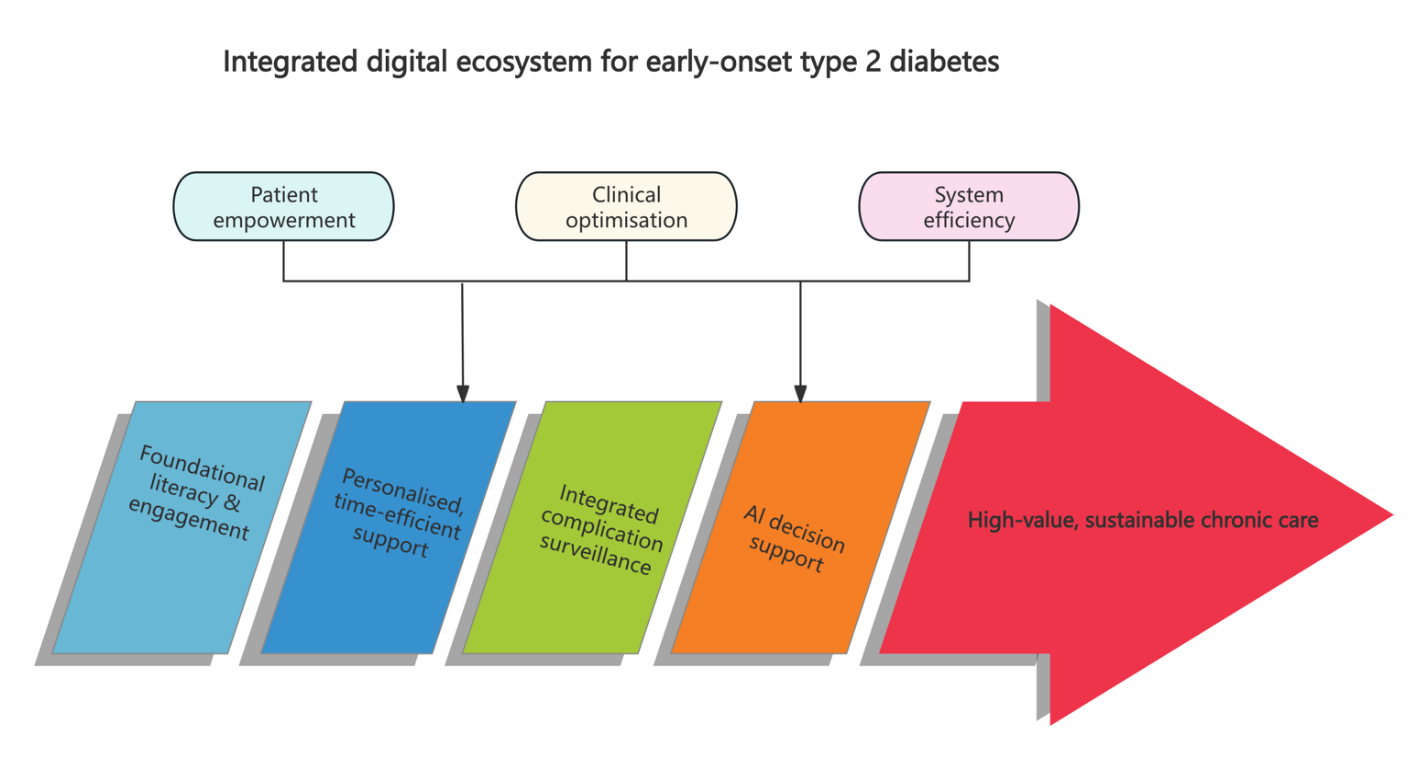


Footnotes: The diagram illustrates an integrated digital ecosystem for people with early-onset type 2 diabetes. Through key modules including foundational health literacy and engagement, personalised high-efficiency support, integrated monitoring of complications, and artificial intelligence (AI)-driven decision support, it synergistically promotes patient empowerment, clinical optimisation, and system efficiency. This leads to high-value, sustainable long-term type 2 diabetes care.
